# Supplementary material for: Co-Culturing Seaweed with Scallops Can Inhibit the Occurrence of Vibrio by Increasing Dissolved Oxygen and pH
Source: Plants (Basel). 2025 Jan 23;14(3):334. doi: 10.3390/plants14030334 (PMC11820688; doi:10.3390/plants14030334)
Supplement: Supplementary file 1 [file plants-14-00334-s001.zip › Supplementary Materials1-Figure S and Table S.pdf]

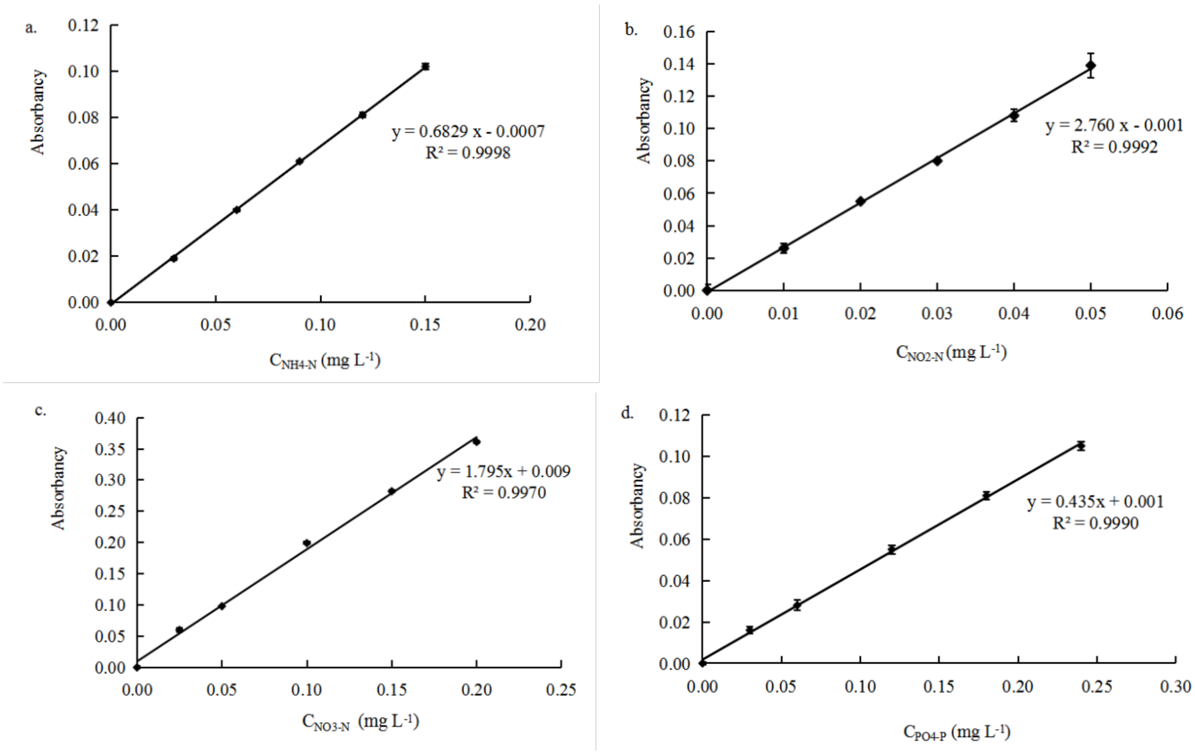

Figure S1 The standard curves of NH_4-N (**a**), NO_2-N (**b**), NO_3-N (**c**), and PO_4-P (**d**) in Experiment D. The values are mean ± SD and the number of independent samples are 3.

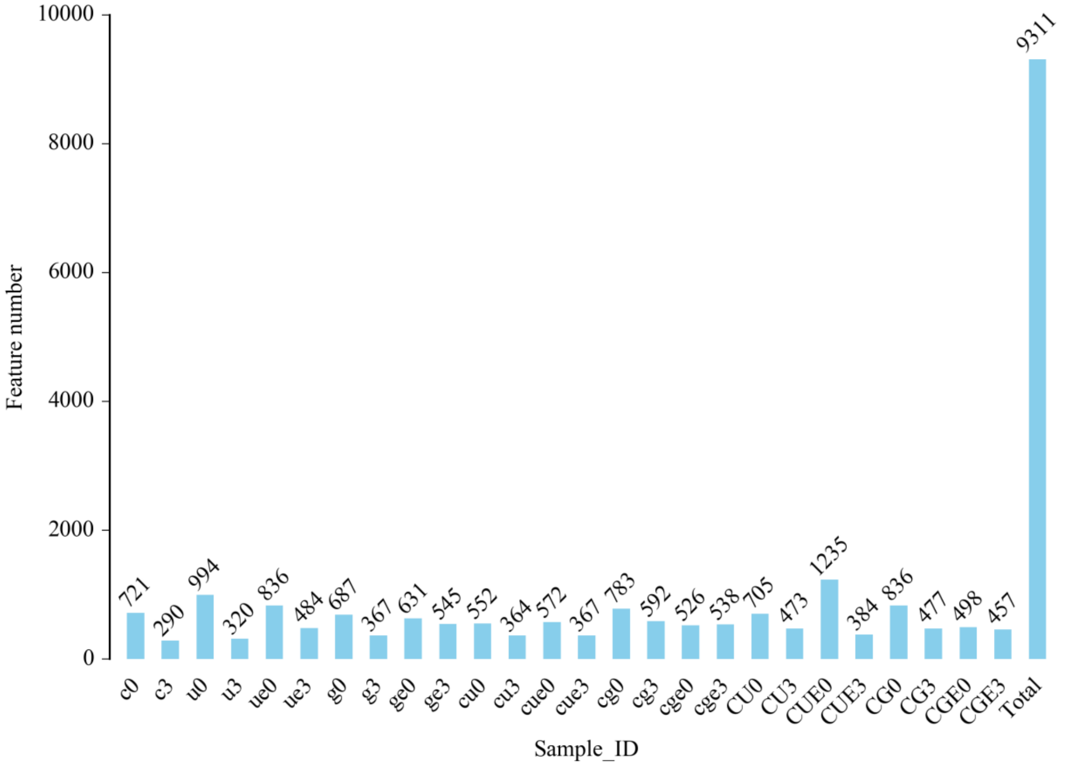

Figure S2 Distribution of the number of ASVs for 26 microbial samples from seawater and algal epiphytes

Table S1 The PCR reaction contents for amplifying the V3-V4 region

**Components**	**Volume**
DNA template	5-50 ng
Forward primer (10μM)	0.3 μl
Reverse primer (10μM)	0.3 μl
KOD FX Neo Buffer	5 μl
dNTP (2 mM each)	2 μl
KOD FX Neo	0.2 μl
ddH_2O	up to 20μL

Table S2 Acronyms corresponding to the sample of microbial diversity in Experiment D

**Treatment**	**Cultured species (Symbol)**	**Seawater environment**		**Epiphytic to alga**	
		**Before culture**	**After culture**	**Before culture**	**After culture**
Control groups	*C. farreri* (cm）	c0	c3	-	-
	U. pertusa (um*)*	u0	u3	ue0	ue3
	G. lemaneiformis (gm)	g0	g3	ge0	ge3
	C. farreri + *U. Pertusa* (cu)	cu0	cu3	cue0	cue3
	C. farreri + *G. lemaneiformis* (cg)	cg0	cg3	cge0	cge3
Experimental groups	*C. farreri* + *U. pertusa* (CU)	CU0	CU3	CUE0	CUE3
	C. farreri + *G. lemaneiformis* (CG)	CG0	CG3	CGE0	CGE3
